# Neutrophil-to-Lymphocyte Ratio, Mediterranean Diet, and Bone Health in Coeliac Disease Patients: A Pilot Study

**DOI:** 10.1155/2019/7384193

**Published:** 2019-06-20

**Authors:** Francesca Palmacci, Elisabetta Toti, Anna Raguzzini, Giovina Catasta, Paola Aiello, Ilaria Peluso, Mariangela Biava, Maura Palmery

**Affiliations:** ^1^Department of Chemistry and Drug Technologies, University “La Sapienza” Rome, Italy; ^2^Research Centre for Food and Nutrition, Council for Agricultural Research and Economics (CREA-AN), Rome, Italy; ^3^Department of Physiology and Pharmacology “V. Erspamer”, Sapienza University of Rome, Italy; ^4^Universidad Católica San Antonio de Murcia (UCAM), Murcia, Spain

## Abstract

Neutrophil-to-lymphocyte ratio (NLR) has been proposed as a bone loss index in postmenopausal women and as a marker of inflammation in coeliac patients. The aims of this work were to evaluate the effect of gluten-free diet (GFD) on NLR retrospectively and study the relationship between NLR and Mediterranean diet adherence and selected food groups (fruits, vegetables, red meat, potatoes, and unrefined and refined cereals). Adult individuals (*n* = 50), who had been on a strict GFD by at least 6 months, were recruited. The degree of adherence to the Mediterranean diet was calculated with two different scores: the Mediterranean Diet Score (MDS-14), assessed through the validated 14-item questionnaire of the PREDIMED study, and the MEDScore (Score-55) proposed by Panagiotakos. The latter includes the consumption of unrefined cereals (UC). High percentages of osteopenia and osteoporosis were found within the recruited subjects, who furnished the reports of bone mineral density (BMD), in particular in postmenopausal (Post-M) women. Recent NLR was higher in subjects with osteoporosis compared to osteopenia and normal BMD. However, retrospective analysis showed both increase and decrease in NLR after GFD, with no significant differences between Marsh grade, anemia, and BMD status. Moreover, premenopausal previous pregnancy (Pre-MPP) and Post-M had higher NLR at diagnosis compared to Men and premenopausal (Pre-M), but higher differences were observed in recent NLR between Pre-MPP and Men only. Chocolate consumption was associated with lower recent NLR, whereas the latter was correlated with Score-55, but not with MDS-14. Moreover, refined cereal consumption was correlated with recent NLR. Although large prospective studies are needed in order to clarify the relationship between UC and NLR in coeliac patients, in this pilot study, we have investigated for the first time the relationship between NLR, dietary habit, and osteoporosis in coeliac disease.

## 1. Introduction

Neutrophil-to-lymphocyte ratio (NLR), calculated from complete white blood cell (WBC) count with differential, is an emerging, inexpensive, easy to obtain, widely available marker of inflammation particularly in cancer patients [1–3] and also in subjects with other diseases, including chronic obstructive pulmonary disease [4], acute pulmonary embolism [5], and acute coronary syndrome [6]. Furthermore, NLR has been proposed as a bone loss index in postmenopausal women [7] and as a marker of inflammation in coeliac patients [8, 9].

Despite gluten-free diet (GFD) resulted deficient in vitamin D (vit. D) and calcium [10], it improved bone mineral density (BMD) [11–14] and normalized vit. D and parathyroid hormone (PTH) values [15]. However, GFD does not normalize BMD in all patients, even after the recovery of intestinal mucosa, and other bone injury mechanisms in addition to calcium and vitamin D malabsorption, including proinflammatory cytokines, have been suggested to be involved [16].

Longitudinal studies reported improvement of BMD in children [17, 18] with coeliac disease after the initiation of GFD, making the latter particularly relevant in children and adolescents [17–19]. However, it may not be sufficient in elderly patients with late onset of coeliac disease [20].

In diagnosed adult coeliac disease patients, despite GFD significant increased BMD, values still remain markedly low after 1 year in several patients [21]. In this context, there is a less consistent relationship between disease and clinical manifestations in the elderly, due to multiple coexisting conditions [22]. In particular, menopause is among the risk factors to be taken into consideration in the diagnostic and therapeutic approach of coeliac disease in women [23].

On the other hand, the inflammatory status may be involved in the onset of osteoporosis in coeliac disease and could justify the reduction of BMD in patients with histology and serology returned to normal after the GFD [13, 16, 20, 24]. In this context, in the large Moli-sani study, adherence to the Mediterranean (Med) diet has been associated with a lower NLR [25]. Wholegrain cereals, rich in fibre, vitamins, minerals, and other antioxidant bioactive components, are within the Med diet components associated with the lower risk of noncommunicable chronic diseases [26].

On the other hand, although CD can induce liver steatosis [27], the role of GFD on cardiometabolic risk factors, such as obesity, serum lipid levels, and insulin resistance, is controversial, due to the high levels of lipids, sugar, and salt in gluten-free products [28, 29]. GFD was found to be poor in dietary fibre [10, 30], but the effect of fibre on calcium absorption and/or BMD is still debated [31–34]. On the other hand, it has been suggested that a low fibre intake may predispose coeliac disease patients to persistent symptoms despite negative antibodies and normal intestinal histology [35]. In this context, a recent randomized clinical trial (RCT) reported increases in vit. D levels in children and adolescents after a prebiotic treatment [36]. Both fibre and polyphenols, contained in plant foods typical of the Med diet, have prebiotic effects [37, 38] and in a recent large retrospective study association between NLR and gut microbiota have been found [39]. Previous studies in Italy reported lower fibre (g/d: 7.3 ± 4.9 vs. 12.8 ± 4.4) [30] and fruit (g/d: 223.5 ± 13.9 vs. 275.0 ± 15.4) [40] intake and higher red meat (g/d: 85.4 ± 3.8 vs. 68.9 ± 4.1) [40] and potatoes (g/d: 40.2 ± 2.9 vs. 26.8 ± 3.2) [40] intake in coeliac subjects. Fibre is contained in both unrefined cereals and other plant foods (fruits and vegetables). On the other hand, bread and pizza are typical Mediterranean foods, regardless of fibre content (refined or unrefined), and previous studies did not find any significant difference in the consumption of carbohydrates [30] and pasta [40]. In the European HELENA study, conducted in adolescents, within Mediterranean food groups, the intake of vegetables was associated with lymphocytes (0.106, *p* < 0.05) but not with WBC count [41], suggesting a potential reduction of NLR.

The aims of this work were to evaluate the effect of GFD on NLR retrospectively and study the relationship between NLR and Med diet adherence and selected Mediterranean food groups (fruits, vegetables, red meat, potatoes, and unrefined and refined cereals).

## 2. Materials and Methods

### 2.1. Recruitment, Data Collection, and Selection of the Subjects

Adult individuals (*n* = 50, 9 men and 41 women), who had been on a strict GFD by at least 6 months, were recruited (May-October 2018) by pharmacies and verbal disclosures.

The subjects enrolled in the study were asked to provide diagnostic reports, bone densitometry, and blood analysis, in particular, WBC count. Furthermore, additional data useful to characterize the subjects were gathered through questionnaires and included age, sex, body weight (kg), height (m), heart rate (HR, beats/min.), systolic (SBP) and diastolic (DBP) blood pressure (mmHg), medical history, postmenopausal state and previous pregnancy and/or spontaneous abortion (for women), use of drugs, supplements and special foods in addition to gluten free (i.e., lactose-free), physical activity level (PAL), hours/day of exposure to sunlight, smoking habits, and consumption of alcoholic beverages, cocoa, coffee, tea, and herbal infusions. Participants were also asked to indicate their usual frequency of eating out of home (EOH) at fast food (EOH-F) or other EOH (EOH-R: restaurant, EOH-P: pizzeria, and EOH-B: bar).

The body mass index (BMI) was calculated dividing body weight (in kg) by squared height (in meter). The physical activity level (PAL) was classified as low, moderate, and high using the International Physical Activity Questionnaire (IPAQ) [42].

**Figure 1:**
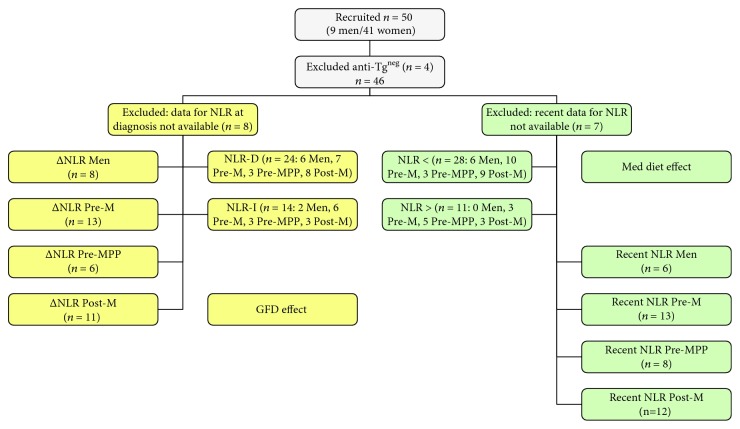
Flow chart of subject's selection for GFD and Med diet effects. AntiTg^neg^: negative for antitransglutaminase antibody at diagnosis; NLR: neutrophil-to-lymphocyte ratio; GFD: gluten-free diet; Med: Mediterranean; Pre-M: premenopausal; Pre-MPP: premenopausal previous pregnancy; Post-M: postmenopausal; ΔNLR: delta NLR after GFD versus NLR before GFD; D: decrease; I: increase. < below cut off and > over cut off 2.32.

In order to evaluate the relationship between diet and NLR, the following criteria were applied. Exclusion criteria included negative antitransglutaminase (Anti-Tg) at diagnosis. Inclusion criteria included availability of data for NLR calculation (i.e., neutrophil and lymphocyte counts within normal ranges). In order to evaluate the effect of GFD, delta NLR after GFD versus NLR before GFD (ΔNLR) was calculated only when WBC count at diagnosis was available and subjects were also classified for increase (NLR-I) or decrease (NLR-D) of NLR after GFD (Figure 1). On the other hand, only subjects who furnished recent (i.e., within a year) complete WBC counts were included in the evaluation of the effect of adherence to Med diet on NLR (Figure 1), and these patients were also grouped on the basis of the cut off value (< below cut off and > over cut off: 2.32) suggested by Sarikaya et al. [8].

### 2.2. Adherence to Mediterranean Diet Analysis

The degree of adherence to the Med diet was calculated with two different scores: the Mediterranean Diet Score (MDS-14), assessed through the validated 14-item questionnaire of the PREDIMED study [43], and the MEDScore (Score-55) proposed by Panagiotakos [44]. The latter, unlike the first, includes the consumption of unrefined cereals (UC). Moreover, MDS-14 and Score-55 differ also for other components. Therefore, we classified the separate subgroups that make up the MDS-14 and Score-55: coherent (CO), incoherent (IC), and different (D) as described in Table 1. In order to evaluate the contribution of each subgroup, percentages of self MDS-14 and self Score-55 were also calculated (MDS-7CO % self 14, MDS-2IC % self 14, MDS-5D % self 14, Score-30CO % self 55, Score-10IC % self 55, and Score-15D % self 55). In the light of previous evidence [30, 40], subscores and % self for fruits, vegetables, red meat, potatoes, and UC were evaluated separately (MDS-1F, MDS-1V, MDS-1RM, Score-5F, Score-5V, Score-5RM, Score-P, Score-5UC, and their % self scores) (Table 1).

Gluten-free whole unrefined cereals/pseudocereals include buckwheat, quinoa, sorghum, millet, and amaranth flours [45]; however, consumers still show a preference to refined, low fibre cereals.

Therefore, a Score-60 was reached by also adding gluten-free refined cereals (RC, score range 0-5 as for UC), being typical of Med diet [30, 40]. The percentages of Score-60 subgroups were calculated based on self Score-60 (% self 60).

### 2.3. Statistical Analysis

The sample size of this pilot study was chosen on the basis of other studies [30, 46–50].

Categorical variables were expressed as percentages [51] and continuous variables were expressed as means with standard error mean (SEM). Results showing a normal pattern were analyzed by analysis of variance (ANOVA), others by Kruskal-Wallis one-way analysis of variance on ranks. The significance of the differences between treatments within the same time and those between the different times within the same treatment group was evaluated using Student-Newman-Keuls (normality test Shapiro-Wilk passed) and Dunn's (normality test Shapiro-Wilk failed) methods. The correlations (Spearman correlation) were analyzed among the parameters of interest.

## 3. Results

### 3.1. Subject Characteristics and Retrospective Analysis

Groups had similar disease durations, despite the differences in age between premenopausal (Pre-M) and postmenopausal (Post-M) (Table 2). Age was related to BMI (0.308, *p* < 0.05), SBP (0.586, *p* < 0.001), and DBP (0.402, *p* < 0.01). Percentages of overweight, underweight, hypertension (SBP > 129/DBP > 84), hypotension (SBP < 90/DBP < 60), dyslipidemia (high cholesterol/LDL or low HDL), and hyperglycemia (fasting blood glucose > 110) were different between groups (Table 2). Percentages of patients with other diseases have been also presented in Table 2, and all subjects were under drug treatment according to the disease. Higher percentages of other diseases, spontaneous abortions, and allergy were reported by Post-M (Table 2). On the other hand, some subjects declared lactose intolerance and/or lactose-free milk consumption (Table 2) and one subject legumes' intolerance.

All subjects, according to selection criteria, had positive anti-Tg (Table 2) at diagnosis, and decreases in anti-Tg were always observed after GFD, as well as negativization of antiendomysial and antigliadin antibodies, when data were available (71% antiendomysial evaluated and 91% antigliadin evaluated).

Concerning the histological evaluation of the duodenal mucosa, the distribution of subjects in Marsh (I, II, and III) classification at diagnosis is reported in Table 2, but in some cases, the Marsh classification was not used.

High percentages of osteopenia and osteoporosis (Table 3) were found within the recruited subjects, who furnished reports of BMD (*n* = 36 of 46), according to a previous study conducted in Italy [52], and a higher percentage of osteoporosis was found in post-M (Table 3). The majority of the subjects had a high or moderate PAL, whereas the percentage of smokers was higher in men (Table 3). On the other hand, a common feature was anemia at diagnosis, not always completely reversible following the GFD (Table 3). However, no differences were found in T-score for smoking habit (smokers -1.1 ± 0.5, nonsmokers -1.8 ± 0.2) or anemia (anemia at diagnosis -1.4 ± 0.3, no anemia at diagnosis -2.0 ± 0.3; anemia at GFD -1.7 ± 0.2, no anemia at GFD -1.7 ± 0.5). On the other hand, age was inversely correlated with T-score (-0.400, *p* < 0.05) and related to hours spent outdoors (autumn/winter, 0.290, *p* = 0.05; spring/summer 0.409, *p* < 0.01).

**Figure 2:**
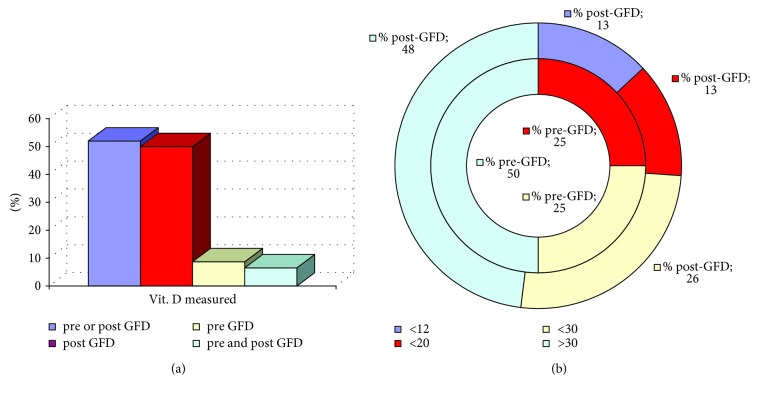
Vitamin D. (a) Percentages of measures before/after gluten-free diet. (b) Percentages within different cut off.

Overall, only half of the subjects furnished reports of vit. D analysis and the majority after GFD (Figure 2(a)).  Figure 2(b) reports the distribution of these values with the cut off levels, recently discussed, that progressively increased from 12 to 20 ng/ml and, finally, to 30 ng/ml [53]. Percentages of subjects under osteoporosis treatment and under supplementation with vit. D are shown in Table 3. Only 3 patients (1 man and 2 women, of whom only one supplemented with vit. D) furnished data of vit. D before and after GFD, and decreases after GFD were observed in these cases (-2.2, -6.1, and -25.0 ng/ml).

On the other hand, recent NLR was higher in subjects with osteoporosis compared to osteopenia and normal BMD, whereas no differences were found in relation to Marsh grade and anemia (Table 4).

Recent NLR was highly correlated with NLR at diagnosis (0.593^∗∗∗^) and less with ΔNLR (0.384^∗^), whereas the latter was inversely related to NLR at diagnosis (-0.443^∗∗^).

Retrospective analysis showed both positive and negative values for ΔNLR, with no significant differences between Marsh grade, anemia, BMD status (Table 4), and groups (Table 5). On the contrary, differences between groups were observed both in recent and at diagnosis NLR (Table 5). Premenopausal previous pregnancy (Pre-MPP) and Post-M had higher NLR at diagnosis compared to Men and Pre-M, but higher differences were observed in recent NLR between Pre-MPP and Men only (Table 5). These data are in line with percentages below and over the cut off suggested by Sarikaya et al. [8], as well as with percentages of increase and decrease NLR (Table 5).

### 3.2. NLR, Dietary Habits, and Adherence to Med Diet

Percentages of subjects with EOH habits and percentages of consumers of chocolate, black tea, green tea, herbal infusion, coffee, and alcoholic beverages (excluding wine, included in scores of adherence to Med diet) were different between Men, Pre-M, Pre-MPP, and Post-M (Table 6). Two-way ANOVA was performed by using dietary habit and NLR cut off value as factors. Among subjects' analysis with NLR over the cut off value, recent NLR was higher in subjects who did not have the habit of eating at the bar and lower in chocolate consumers (Table 6). Chocolate consumption was associated with lower recent NLR even among those with NLR under the cut off value (Table 6).

On the other hand, recent NLR was correlated with Score-55 and Score-60 but not with MDS-14 (Table 7). Concerning the score subgroups and their contribution to the adherence to Med diet, only Score-30CO and Score-5UC (and its % self 55 and self 60) were correlated with recent NLR (Table 7). Score-5UC, Score-30CO, Score-60, MDS-14, MDS-7CO, MDS-2IC, and Score-5RM were all inversely related to years at GFD (Table 7). The latter was correlated with MDS-5D % self 14 and self Score-5F and Score-5V (both % self 55 and % self 60) (Table 7). On the other hand, MDS-14, MDS2IC (and its self 14), and Score-5RM and its % self 55 and self 60 were correlated with age at diagnosis, whereas for MDS-5D % self 14 the correlation is inverse (Table 7). Score-5RM and its % self 60 were correlated with age. Despite MDS-14 and Score-55 were correlated (0.576, *p* < 0.001), only the former was correlated with age and in particular the MDS-2IC (and its % self 14) component (Table 7). MDS-2IC self 14 was significantly lower in Pre-M (Table 8), and this group had also lower consumption of RC compared to Pre-MPP (Table 8) and higher MDS-7CO % self 14 and MDS-1F self 14 compared to Men and Post-M (Table 8).

## 4. Discussion

From 50 subjects, after the selection for anti-Tg at diagnosis, we retrospectively evaluated 46 adult-diagnosed (Table 2) coeliac disease patients at GFD. In this study, we evaluated NLR, being a marker of inflammation in coeliac disease [8, 9] related to postmenopausal osteoporosis [8, 54].

Neither Marsh classification nor anemia was related to recent NLR (Table 5) or T-Score, and the latter also has no significant affected by smoking habit, whereas was correlated with age.

A recent interest is born on the role of some genetic polymorphisms in iron-deficiency anemia- (IDA-) persistent CD at GFD [55]. On the other hand, there is evidence that BMD does not return to normal in coeliacs diagnosed in adulthood and that this finding could be due to menopausal status in women [23] or inflammatory status [24]. In a case report of a 65-year-old postmenopausal woman, hormone replacement therapy, in addition to GFD and supplementation, was unable to inhibit the reduction of BMD and was therefore prescribed oral ibandronate [56]. In the present study, the use of drugs for osteoporosis has been reported only by some Post-M (Table 3). The use of other drugs was in line with comorbidities (Table 2). In particular, as previously reported [52, 57], autoimmune thyroiditis and type 1 diabetes mellitus are common among coeliac patients. In this context, it has been suggested a synergistic effect of hyperglycemia and coeliac autoimmunity on low BMD [58]. Although Post-M presented more comorbidities (Table 2) and osteoporosis (Table 3) and, overall, subjects with osteoporosis had higher recent NLR compared to patients with osteopenia or normal BMD (Table 4), as previously reported [54], Pre-MPP had higher recent NLR (Table 5).

Retrospective analysis revealed that both Pre-MPP and Post-M had higher NLR at diagnosis compared to Men and Pre-M (Table 5). However, percentages of subjects with decrease in NLR after GFD were lower in Pre-MPP compared to Men, Pre-M, and Post-M (Table 5).

Moreover, a high percentage of patients with recent NLR over the cut off value, suggested by Sarikaya et al. [8], have been observed in Pre-MPP compared to other groups. In a study conducted on premenopausal women, low BMD values were associated with elevated serum levels of receptor activator of nuclear factor- (NF-) kappaB (RANK), in the group of coeliac patients compared to controls, despite the normal values of calcium and PTH [24]. Furthermore, in a case report, autoantibodies against osteoprotegerin (OPG), a member of the tumor necrosis factor receptor family that inhibits RANK, were found in a man with coeliac disease and severe osteoporosis not reversible with GFD [59]. However, Larussa and coworkers [60] did not find circulating antibodies against OPG in the serum of 30 celiac patients, regardless of BMD, duodenal histology, and HLA status.

Despite a recent guideline suggested that vit. D levels should be measured at diagnosis and that supplementation with calcium and vit. D should be provided according to its level [58], only half of the subjects furnished reports of vit. D analysis and the majority after GFD (Figure 2(a)). Although only 13% of cases had severe D-hypovitaminosis (Table 3), there is a consensus that levels lower than 20 ng/ml are associated with osteoporosis and the Italian Association of Clinical Endocrinologists recommend to maintain vit. D levels above 30 ng/ml in subjects with malabsorption syndrome, osteopenia, osteoporosis, and under osteoporosis treatment [53].

In the NU-AGE study, conducted in elderly people, a Med diet with a vit. D supplement (1 year) reduced the rate of bone loss in individuals with osteoporosis [61] but increased the TLR-stimulated ex vivo expression of the costimulatory molecules CD40 and CD86 in women with a BMI < 31. The authors suggested that this gender-effect may be related to the fact that women exhibit stronger cellular- and humoral-mediated immune responses compared to men, with a higher risk of autoimmune disease [62].

Overlap exists between coeliac disease and other gut disorders, such as fermentable oligosaccharides, disaccharides, monosaccharides, and polyols (FODMAPs), and lactose intolerance [63]. However, in our study, subjects who declared lactose intolerance also declared to consume lactose-free milk. Moreover, all subjects, according to the selection criteria, had anti-Tg (Table 2) at diagnosis, and decreases in anti-Tg were always observed after GFD, as well as negativization of antiendomysial and antigliadin antibodies. Concerning FODMAP, a recent retrospective study [64] reported that coeliac disease patients, despite the low intake of gluten-free cereals high in FODMAP, consumed a significant amount of fruits and vegetables high in FODMAP. Therefore, the authors suggested that a low-FODMAP diet should be a supportive therapy in subjects at GFD. In agreement, a recent RCT [65] reported that in subjects on GFD with persistence of functional gastrointestinal disorders, a diet low in FODMAP reduced abdominal pain and improved the fecal consistency.

In our study, only Pre-M had higher MDS-7CO % self 14 and MDS-1F self 14, compared to Men and Post-M (Table 8), despite only in Pre-PP recent NLR mean was over the cut off levels (Table 5).

However, overall recent NLR was correlated with Score-55, Score-60 (including Score-5RC), Score-30CO (high consumption of olive oil, fruits, vegetables, legumes, and fish and low consumption of red meat), and Score-5UC (and its % self 55 and self 60) (Table 7). Looking to the differences in other dietary habits, the Pre-MPP group presented lower percentages of subjects with EHO-B and of chocolate consumers; the two factors for which an interaction with the relationship was found (Table 6). On the one hand, these differences can affect the dissimilarities in recent NLR (Table 5) but, on the other hand, they must be taken into account when evaluating the two-way ANOVA with dietary habits and cut off as factors (Table 6). Keeping in mind these considerations, interesting chocolate consumption was associated with lower recent NLR regardless the cut off level (Table 6).

In this context, in a randomized double-blind crossover study [66] in overweight men (age 45-70 yr), after 4 weeks of consumption, delta lymphocytes' count versus baseline levels were −0.05 ± 0.19 and −0.03 ± 0.2 n/nl for high flavanol chocolate (HFC: 70 g contained 1078 mg flavanols, of which 349 mg epicatechins) and normal flavanol chocolate (NFC: 70 g contained 259 mg flavanols, of which 97 mg epicatechins), respectively. Delta neutrophils' count versus baseline levels were −0.30 ± 0.70 and −0.0 ± 0.8 n/nl for NFC and HFC, with no differences between treatments.

Our study presents some limitations such as the small number of subjects enrolled and the absence of complete data in some cases (in particular for vit. D levels), due to the retrospective nature of the study. Moreover, due to the high percentage of nonclassified histological evaluations (n.c., Table 2), we cannot observe the previously reported correlation between BMD and Marsh stage at diagnosis [48]. However, the major limitation of our study is that no data were available concerning anti-Saccharomyces cerevisiae antibodies (ASCA). It has been suggested that the high prevalence of ASCA in coeliac disease may be the effect of a nonspecific immune response in the course of chronic small bowel disease [67], and it has been reported that serum levels of ASCA correlated with the grade of mucosal morphology, as the ASCA serum levels declined in accordance with mucosal healing [68]. ASCA (immunoglobulin G and/or immunoglobulin A) were frequently observed during active coeliac disease and decreased after GFD [68–71], but more in children than in adults [70–72], who resulted more frequently ASCA positive at diagnosis [71, 72]. After successful adherence to a GFD and normalization of anti-Tg 29% of adults remained ASCA positive, 7% of whom remained positive for both IgA and IgG ASCA [71]. It has been suggested that this finding can be explained by the well-known fact that gut permeability normalizes much better in children than in adults [71]. Significantly higher faecal counts of Saccharomyces were found in patients reporting persistent symptoms, despite the GFD, compared to noncoeliac controls [73]. Although further studies are needed in order to investigate the relationship between NLR levels over the cut off and ASCA, in the present study, no interactions were found between EOH-P and NLR cut off by two-way ANOVA (Table 6). Moreover, recent NLR was correlated with UC (Table 7), but not RC, consumption.

## 5. Conclusion

In this pilot study, we have investigated for the first time the relationship between NLR, dietary habit, and osteoporosis in coeliac disease. Although recent NLR was higher in subjects with osteoporosis compared to osteopenia and normal BMD, retrospective analysis showed both increase and decrease in NLR after GFD, with no significant differences between Marsh grade, anemia, and BMD status. Refined cereal consumption was correlated with recent NLR. However, despite no data were available concerning ASCA, due to the retrospective nature of our study, in the present study, no relationships were found between eating out of home at pizzeria or gluten-free refined cereal consumption and NLR. On the other hand, recent NLR was correlated with Score-55, including the consumption of UC, but not with MDS-14.

In conclusion, more prospective studies are needed in order to clarify the relationship between UC and NLR in coeliac patients since coeliac patients are generally encouraged to rise intakes of dietary fibre through the increase in the consumption of whole-grain and enriched/fortified gluten-free flours, breads, pasta, and cereals whenever possible.

## Figures and Tables

**Table 1:** MDS-14, Score-55, and Score 60 subgroups and self-scores.

	Score range	High score for high consumption	High score for low consumption
MDS-7CO (MDS-14 subgroup)	0-7	Olive oil, fruits, vegetables, legumes, and fish	Red meat
Score-30CO (Score-55 subgroup)	0-30	Olive oil, fruits, vegetables, legumes, and fish	Red meat
MDS-2IC (MDS-14 subgroup)	0-2	Wine and white meat	
Score-10IC (Score-55 subgroup)	0-10		Wine and white meat
MDS-5D (MDS-14 subgroup)	0-5	Nuts and Med-sauce	Butter, carbonated beverages, and sweets
Score-15D (Score-55 subgroup)	0-15	UC and potatoes	Full dairy products
Score-5UC	0-5	0 = never	
		1 = 1-4/month	
		2 = 5-8/month	
		3 = 9-12/month	
		4 = 13-18/month	
		5 > 18/month	
Score-5RC (Score-60 = Score-55 + Score 5RC)	0-5	As for 5UC	
MDS-1F	0-1	1 ≥ 3 units/d	
Score-5F	0-5	As for 5UC	
MDS-1V	0-1	1 ≥ 2 servings/d (1 serving: 200 g)	
Score-5V	0-5	As for 5UC	
MDS-1RM	0-1	1 < 1 serving/d (1 serving: 100-150 g)	
Score-5RM	0-5	0 > 18/month	
		1 = 13-18/month	
		2 = 9-12/month	
		3 = 5-8/month	
		4 = 1-4/month	
		5 = never	
Score-5P	0-5	As for 5UC	
% self 14	0-100	(Subgroup score/total self MDS − 14)∗100	
% self 55	0-100	(Subgroup score/total self − Score − 55)∗100	
% self 60	0-100	(Subgroup score/total self − Score − 60)∗100	

CO: coherent; IC: incoherent; DF: different; Med-sauce: Mediterranean sauce made with tomato, olive oil, garlic, or onion; UC: unrefined cereals; RC: refined cereals; F: fruits; V: vegetables; RM: red meat; P: potatoes.

**Table 2:** Characteristics of subjects at GFD.

	Men	Pre-M	Pre-MPP	Post-M^#^
Age (years)	37.6 ± 5.5	29.6 ± 2.9^∗^	43.2 ± 2.0	54.5 ± 2.0^∗^
Years to diagnosis	6.6 ± 1.9	6.0 ± 1.6	6.2 ± 1.9	6.8 ± 1.6
Age years at diagnosis	31.0 ± 6.6	23.6 ± 3.2^∗^	37.0 ± 2.2	47.7 ± 2.5^∗^
Anti-Tg U/ml at diagnosis	722 ± 606	70 ± 18	239 ± 98	93 ± 18
Marsh at diagnosis				I 7.7%
	II 11.1%			II 7.7%
	III 77.8%	III 80.0%	III 77.8%	III 53.8%
	nc 11.1%	nc 20.0%	nc 22.2%	nc 30.8%
BMI (kg/m^2^)	23.0 ± 0.8	21.8 ± 0.5	22.2 ± 0.6	23.4 ± 0.7
Overweight	22.2%	—	11.1%	15.4%
Underweight	—	13.3%	—	—
SBP (mmHg)	113.9 ± 3.2	106.8 ± 2.9	115.6 ± 1.8	118.2 ± 3.3
DBP (mmHg)	75.0 ± 3.1	63.7 ± 2.8^∗^	80.0 ± 0.9^∗^	74.8 ± 2.4
HR (beat/min.)	65.8 ± 2.0	64.2 ± 2.3	69.8 ± 3.4	64.9 ± 2.4
Hypertension	22.2%	—	11.1%	30.8%
Use of drugs for hypertension			11.1%	7.7%
Hypotension	—	33.3%	—	7.7%
Hyperglycemia	—	—	—	23%
Use of drugs for hyperglycemia				23.1%
Dyslipidemia	22.2%		22.2%	
Use of drugs for dyslipidemia				7.7%
Other diseases	11.1% atopic dermatitis	5% autoimmune diseases^^^	11.1% autoimmune diseases^†^	30.8% autoimmune diseases^‡^
				7.7% atopic dermatitis
				7.7 % COPD
Spontaneous abortions		6.6%	22.2%	30.8%
Allergy (nickel, pollen, etc.)	11.1%	26.6%	11.1%	53.8%
Lactose intolerance	22.2%	13.3%	11.1%	15.4%

GFD: gluten-free diet; Pre-M.NPP: premenopausal no previous pregnancy; Pre-MPP: premenopausal previous pregnancy; Post-M: postmenopausal. ^#^years of menopausal state 8.1 ± 1.3. BMI: body mass index; SBP: systolic blood pressure; DBP: diastolic blood pressure; HR: heart rate; ^^^Hashimoto thyroiditis (3), psoriatic arthritis (1), vitiligo (1). ^†^Antiphospholipid syndrome (1). ^‡^Hashimoto thyroiditis (3), type 1 diabetes (2), lupus (1); COPD: chronic obstructive pulmonary disease. Data are expressed as mean ± SEM or percentages. ^∗^*p* < 0.05.

**Table 3:** GFD, anemia, osteoporosis, and lifestyle.

	Men	Pre-M	Pre-MPP	Post-M
Anemia at GFD	22.2%	21.4%	11.1%	7.7%
Anemia at diagnosis	33.3%	50.0%	62.5%	33.3%
Supplements for anemia				
Iron	11.1%	13.3%	33.3%	
Folic acid		13.3%		7.7%
BMD	*n* = 6	*n* = 9	*n* = 8	*n* = 13
Normal	—	33.3%	12.5%	15.4%
Osteopenia	66.7%	66.7%	87.5%	30.8%
Osteoporosis	33.3%	—	—	53.8%
T-Score	−2.0 ± 0.3	−1.0 ± 0.1^∗^	−1.9 ± 0.3	−2.1 ± 0.4^∗^
BMD years from diagnosis	5.3 ± 2.3	2.9 ± 1.2	5.8 ± 1.8	5.4 ± 1.9
Use of drugs for osteoporosis				23.1%
Vit. D supplement	22.2%	33.3%		53.8%
Hours spent outdoors				
(Autumn/winter)	3.7 ± 1.1	2.2 ± 0.3	1.4 ± 0.7	2.3 ± 0.4
(Spring/summer)	5.9 ± 1.1	4.7 ± 0.5	4.4 ± 0.4	6.4 ± 0.7
High PAL	77.8%	80.0%	77.8%	69.2%
Moderate PAL	22.2%	13.3%	22.2%	30.8%
Smokers	55.5%	6.7%	11.1%	23.1%

GFD: gluten-free diet; Pre-M.NPP: premenopausal no previous pregnancy; Pre-MPP: premenopausal previous pregnancy; Post-M: postmenopausal. BMD: bone mineral density; PAL: physical activity level. Data are expressed as mean ± SEM or percentages. ^∗^*p* < 0.05.

**Table 4:** NLR, coeliac disease, anemia, and BMD.

	Recent NLR	NLR at diagnosis	ΔNLR
Marsh I	2.6 ± 0.7	2.3 ± 0.5	−0.7 ± 0.6
Marsh II	2.2 ± 0.5	1.8 ± 0.4	−0.7 ± 0.4
Marsh III	2.5 ± 0.1	2.4 ± 0.1	0.0 ± 0.1
n.c. Marsh	2.6 ± 0.3	2.1 ± 0.2	0.1 ± 0.2

Anemia at diagnosis	Yes: 2.6 ± 0.2	Yes: 2.3 ± 0.1	Yes: 0.2 ± 0.1
	No: 2.3 ± 0.2	No: 2.2 ± 0.1	No: −0.2 ± 0.1

Anemia after GFD	Yes: 2.1 ± 0.3	Yes: 2.4 ± 0.2	Yes: 0.1 ± 0.2
	No: 2.6 ± 0.1	No: 2.3 ± 0.1	No: −0.1 ± 0.2

BMD normal	2.1±0.2^∗∗^	2.7 ± 0.2	−0.3 ± 0.3
Osteopenia	2.5 ± 0.1^§^	2.1 ± 0.1	0.0 ± 0.1
Osteoporosis	3.5±0.3^∗∗§^	2.5 ± 0.2	−0.2 ± 0.2

NLR: neutrophil-to-lymphocyte ratio; BMD: bone mineral density; GFD: gluten-free diet; ΔNLR: delta NLR after GFD versus NLR before GFD. Data are expressed as mean ± SEM. ^§^*p* < 0.05; ^∗∗^*p* < 0.01.

**Table 5:** NLR.

	Men	Pre-M	Pre-MPP	Post-M
Recent NLR	1.3 ± 0.2^∗^	1.8 ± 0.2	3.2 ± 0.5 (8)^∗^	1.9 ± 0.3
> (3.4 ± 0.2)	—	23.1%	62.5%	18.2%
< (1.5 ± 0.1)	100.0%	76.9%	37.5%	81.8%
NLR at diagnosis	1.7 ± 0.3^∗^	1.7 ± 0.2^∗^	2.7 ± 0.3^∗^	2.7 ± 0.3^∗^
> at diagnosis (3.0 ± 0.1)	25.0%	23.1%	66.6%	63.6%
< at diagnosis (1.6 ± 0.1)	75.0%	76.9%	33.3%	36.4%
ΔNLR	−0.4 ± 0.2	−0.2 ± 0.2	0.3 ± 0.5	−0.7 ± 0.4
NLR-I (0.7 ± 0.2)	25.0%	38.5%	50.0%	27.3%
NLR-D (−0.8 ± 0.1)	75.0%	61.5%	50.0%	72.7%

NLR: neutrophil-to-lymphocyte ratio; ΔNLR: delta NLR after GFD versus NLR before GFD; D: decrease; I: increase. < below cut off and > over cut off 2.32; Pre-M: premenopausal; Pre-MPP: premenopausal previous pregnancy; Post-M: postmenopausal. Data are expressed as mean ± SEM or percentages. ^∗^*p* < 0.05.

**Table 6:** Dietary habits.

	%		NLR recent	Within cut off NLR
Men	66.7	EOH-B	Yes: 2.2 ± 0.2	Yes and NLR>: 2.9 ± 0.3^∗^
Pre-M	60.0		No: 2.6 ± 0.1	No and NLR>: 3.9 ± 0.2^∗^
Pre-MPP	33.3			
Post-M	76.9			

Men	55.5	EOH-R	Yes: 2.5 ± 0.1	
Pre-M	66.7		No: 2.7 ± 0.2	
Pre-MPP	55.5			
Post-M	46.1			

Men	77.8	EOH-P	Yes: 2.4 ± 0.2	
Pre-M	60.0		No: 2.5 ± 0.2	
Pre-MPP	66.7			
Post-M	53.8			

Men	11.1	EOH-F	Yes: 2.2 ± 0.4	
Pre-M	40.0		No: 2.6 ± 0.1	
Pre-MPP	—			
Post-M	—			

Men	55.5	Chocolate	Yes: 2.1 ± 0.1^∗^	Yes and NLR > 2.9±0.3^∗∗^
Pre-M	53.3		No: 2.8 ± 0.1^∗^	No and NLR>: 4.1±0.2^∗∗^
Pre-MPP	22.2			Yes and NLR < 1.2±0.2^∗∗∗^
Post-M	30.7			No and NLR<: 1.6±0.1^∗∗∗^

Men	—	Black tea	Yes: 2.7 ± 0.3	
Pre-M	13.3		No: 2.5 ± 0.1	
Pre-MPP	—			
Post-M	15.4			

Men	—	Green tea	Yes: 2.4 ± 0.3	
Pre-M	13.3		No: 2.6 ± 0.1	
Pre-MPP	11.1			
Post-M	7.7			

Men	—	Herbal infusion	Yes: 2.5 ± 0.2	
Pre-M	20.0		No: 2.6 ± 0.1	
Pre-MPP	44.4			
Post-M	15.4			

Men	55.5	Coffee	≥3: 2.4 ± 0.2	
Pre-M	13.3		≤2: 2.6 ± 0.1	
Pre-MPP	33.3			
Post-M	23.0			

Men	77.8	Alcoholic beverages	Yes: 2.8 ± 0.2	
Pre-M	53.3	(excluding wine)	No: 2.5 ± 0.1	
Pre-MPP	22.2			
Post-M	23.1			

Pre-M: premenopausal; Pre-MPP: premenopausal previous pregnancy; Post-M: postmenopausal; EOH: eating out of home; EOH-B: EOH at bar; EOH-R: EOH at restaurant; EOH-P: EOH at pizzeria; EOH-F: EOH at fast food. Data are expressed as mean ± SEM or percentages. ^∗^*p* < 0.05; ^∗∗^*p* < 0.01; ^∗∗∗^*p* < 0.001.

**Table 7:** Med diet adherence correlations.

	Recent NLR	Age	Years at GFD	Age at diagnosis
MDS-14		0.323^∗^	-0.331^∗^	0.398^∗^
Score-55	0.419^∗∗^			
Score-60	0.469^∗∗^		-0.322^∗^	
MDS-7CO			-0.308^∗^	
MDS-2IC		0.446^∗∗^	-0.335^∗^	0.542^∗∗∗^
MDS-2IC % self 14		0.351^∗^		0.380^∗∗^
MDS-5D				
MDS-5D % self 14			0.346^∗^	-0.322^∗^
Score-30CO	0.334^∗^		-0.322^∗^	
Score-5UC	0.373^∗^		-0.308^∗^	
Score-5UC % self 55	0.340^∗^			
Score-5UC % self 60	0.330^∗^			
Score-5F % self 55			0.475^∗∗∗^	
Score-5F % self 60			0.452^∗∗^	
Score-5V % self 55			0.397^∗∗^	
Score-5V % self 60			0.380^∗∗^	
Score-5P		-0.376^∗^		
Score-5P % self 55				
Score-5P % self 60				
Score-5RM		0.306^∗^	-0.314^∗^	0.365^∗^
Score-5RM self 55				0.335^∗^
Score-5RM self 60		0.302^∗^		0.347^∗^

CO: coherent; IC: incoherent; DF: different; UC: unrefined cereals; F: fruits; V: vegetables; RM: red meat; ^∗^*p* < 0.05; ^∗∗^*p* < 0.01; ^∗∗∗^*p* < 0.001.

**Table 8:** Adherence to Med diet.

	Men	Pre-M	Pre-MPP	Post-M
MDS-14	5.8 ± 0.5	6.3 ± 0.6	5.8 ± 0.4	6.5 ± 0.6
Score-55	28.3 ± 1.1	32.4 ± 1.2	32.2 ± 1.1	31.8 ± 1.1
Score-60	32.7 ± 1.2	35.3 ± 1.0	37.0 ± 1.0	35.8 ± 0.9
MDS-7CO	1.8 ± 0.3	3.0 ± 0.3	2.3 ± 0.3	2.4 ± 0.4
MDS-7CO % self 14	29.6 ± 2.8^∗^	46.8±2.8^∗∗§^	39.9 ± 3.7	34.7 ± 3.1^§^
Score-30CO	19.8 ± 0.6	21.0 ± 0.8	21.4 ± 0.7	21.5 ± 0.5
Score-30CO % self 55	70.0 ± 0.7	65.0 ± 1.1	67.0 ± 2.6	68.0 ± 1.8
Score-30CO % self 60	60.7 ± 1.0	59.5 ± 1.3	58.1 ± 2.0	60.1 ± 1.3
MDS-2IC	1.1 ± 0.3	0.6 ± 0.1	0.9 ± 0.1	1.1 ± 0.1
MDS-2IC % self 14	18.9 ± 4.7^∗^	8.3 ± 1.9^∗§^	15.6 ± 2.1	17.5 ± 2.6^§^
Score-10IC	6.3 ± 0.4	6.8 ± 0.3	6.4 ± 0.4	6.3 ± 0.4
Score-10IC % self 55	22.2 ± 1.0	21.2 ± 1.0	20.3 ± 1.5	19.8 ± 1.0
Score-10IC % self 60	19.3 ± 1.0	19.3 ± 3.1	17.6 ± 1.4	17.6 ± 0.9
MDS-5D	2.9 ± 0.3	2.7 ± 0.3	2.6 ± 0.2	3.1 ± 0.3
MDS-5D % self 14	51.3 ± 4.0	44.9 ± 3.6	44.5 ± 2.4	47.8 ± 3.5
Score-15D	2.2 ± 0.2	4.6 ± 0.7	4.3 ± 1.2	4.1 ± 0.8
Score-15D % self 55	7.8 ± 0.7	13.8 ± 1.7	12.8 ± 3.0	12.2 ± 2.0
Score-15D % self 60	6.7 ± 0.6	12.8 ± 1.6	11.2 ± 2.7	11.0 ± 1.9
Score-5UC	0.7 ± 0.2	2.1 ± 0.5	1.6 ± 0.5	1.5 ± 0.4
Score-5UC % self 55	2.2 ± 0.7	6.0 ± 1.3	4.6 ± 1.2	4.6 ± 1.0
Score-5UC % self 60	1.9 ± 0.7	5.7 ± 1.3	4.0 ± 1.0	4.1 ± 0.9
Score-5RC	4.3 ± 0.4	2.9 ± 0.5^∗^	4.8 ± 0.2^∗^	4.0 ± 0.3
Score-5RC % self 60	13.2 ± 1.2^∗^	8.5 ± 1.4^∗§^	13.0 ± 0.7^§^	11.4 ± 1.1
MDS-1F	0.0 ± 0.1	0.4 ± 0.1	0.2 ± 0.1	0.1 ± 0.1
MDS-1F % self 14	0.0 ± 0.1^∗^	6.4 ± 2.3^∗§^	3.6 ± 2.3	0.7 ± 0.7^§^
Score-5F	4.3 ± 0.2	4.5 ± 0.2	4.6 ± 0.4	4.7 ± 0.2
Score-5F % self 55	16.1 ± 0.6	15.0 ± 0.5	15.0 ± 0.9	15.7 ± 0.6
Score-5F % self 60	13.2 ± 0.4	12.7 ± 0.6	12.4 ± 1.2	13.2 ± 0.6
MDS-1V	0.3 ± 0.2	0.7 ± 0.1	0.4 ± 0.2	0.7 ± 0.1
MDS-1V % self 14	4.7 ± 2.4	9.6 ± 2.0	7.1 ± 2.9	10.0 ± 2.1
Score-5V	4.6 ± 0.2	4.8 ± 0.1	4.8 ± 0.2	4.9 ± 0.1
Score-5F % self 55	15.3 ± 0.5	13.9 ± 0.7	14.3 ± 1.5	15.0 ± 0.8
Score-5F % self 60	13.9 ± 0.4	13.7 ± 0.4	13.0 ± 0.7	13.8 ± 0.4
MDS-1RM	0.1 ± 0.1	0.4 ± 0.1	0.3 ± 0.2	0.2 ± 0.1
MDS-1RM % self 14	1.2 ± 1.2	5.5 ± 1.8	4.8 ± 2.4	2.1 ± 1.5
Score-5RM	2.2 ± 0.4	2.5 ± 0.3	2.8 ± 0.2	2.5 ± 0.3
Score-5F % self 55	8.0 ± 1.4	7.4 ± 0.8	8.7 ± 0.8	7.9 ± 0.8
Score-5F % self 60	7.0 ± 1.2	6.8 ± 0.8	7.6 ± 0.7	7.1 ± 0.8
Score-5P	1.6 ± 0.2	1.5 ± 0.2	1.7 ± 0.3	1.6 ± 0.3
Score-5F % self 55	5.5 ± 0.6	4.8 ± 0.8	5.0 ± 0.8	5.1 ± 0.9
Score-5F % self 60	4.8 ± 0.5	4.3 ± 0.7	4.4 ± 0.7	4.5 ± 0.8

Data are expressed as mean ± SEM. Pre-M: premenopausal; Pre-MPP: premenopausal previous pregnancy; Post-M: postmenopausal; CO: coherent; IC: incoherent; D: different; UC: unrefined cereals; RC: refined cereals; F: fruits; V: vegetables; RM: red meat; P: potatoes. ^§^*p* < 0.05; ^∗^*p* < 0.05; ^∗∗^*p* < 0.01.

## Data Availability

The data used to support the findings of this study are restricted by the Ethics Committee in order to protect patient privacy. Data are available from Ilaria Peluso for researchers who meet the criteria for access to confidential data.
